# Coping Strategies, Quality of Life, and Neurological Outcome in Patients Treated with Mechanical Thrombectomy after an Acute Ischemic Stroke

**DOI:** 10.3390/ijerph17176014

**Published:** 2020-08-19

**Authors:** Silvia Reverté-Villarroya, Antoni Dávalos, Sílvia Font-Mayolas, Marta Berenguer-Poblet, Esther Sauras-Colón, Carlos López-Pablo, Estela Sanjuan-Menéndez, Lucía Muñoz-Narbona, Rosa Suñer-Soler

**Affiliations:** 1 Department of Nursing, Rovira Virgili University, Campus Terres de l’Ebre, 43500 Tortosa, Spain; mberenguer.ebre.ics@gencat.cat (M.B.-P.); clopezp.ebre.ics@gencat.cat (C.L.-P.); 2 Hospital de Tortosa Verge de la Cinta, Pere Virgili Institut, 43500 Tortosa, Spain; esther.sauras@iispv.cat; 3 RETICS Research Group, Department of Neurosciences, Hospital Universitari Germans Trias i Pujol, 08916 Badalona, Spain; adavalos.germanstrias@gencat.cat (A.D.); lmunoz@igtp.cat (L.M.-N.); 4 Quality of Life Institute, University of Girona, 17003 Girona, Spain; silvia.font@udg.edu; 5 Stroke Unit, Hospital Universitari Vall d’Hebron, 08035 Barcelona, Spain; ensayosictus@gmail.com; 6 Department of Nursing, University of Girona, 17003 Girona, Spain; rosa.sunyer@udg.edu

**Keywords:** stroke, endovascular treatment, coping, health behavior, health-related quality of life

## Abstract

New reperfusion therapies have improved the clinical recovery rates of acute ischemic stroke patients (AISP), but it is not known whether other factors, such as the ability to cope, might also have an effect. The aim of this study was to evaluate the effect of endovascular treatment (EVT) on coping strategies, quality of life, and neurological and functional outcomes in AISP at 3 months and 1 year post-stroke. A multicenter, prospective, longitudinal, and comparative study of a sub-study of the participants in the Endovascular Revascularization with Solitaire Device versus Best Medical Therapy in Anterior Circulation Stroke within 8 Hours (REVASCAT) clinical trial was conducted after recruiting from two stroke centers in Catalonia, Spain. The cohort consisted of 82 ischemic stroke patients (*n* = 42 undergoing EVT and *n* = 40 undergoing standard best medical treatment (BMT) as a control group), enrolled between 2013–2015. We assessed the coping strategies using the Brief Coping Questionnaire (Brief-COPE-28), the health-related quality of life (HRQoL) with the EQ-5D questionnaire, and the neurological and functional status using the National Institute of Health Stroke Scale (NIHSS), Barthel Index (BI), modified Rankin Scale (mRS), and Stroke Impact Scale-16 (SIS-16). Bivariate analyses and multivariate linear regression models were used. EVT patients were the ones that showed better neurological and functional outcomes, and more patients presented reporting no pain/discomfort at 3 months; paradoxically, problem-focused coping strategies were found to be significantly higher in patients treated with BMT at 1 year.

## 1. Introduction

The progress of medical treatments and urgent care strategies has evolved in recent decades, which has allowed for the survival rate of stroke patients to improve [1]. In addition, patients’ neurological involvement and functional dependence have been reduced and the life-changing consequences that affected people must cope with after a stroke have been improved [2].

The usual clinical practice has focused on assessing the sequelae after a stroke, but establishing the relationship between these sequelae and the functional impact is not a simple task and is little quantified. Many patients with a stroke will also experience a reduced health-related quality of life (HRQoL) [2]. Known predictors of HRQoL are functional constraints, age, sex, socioeconomic status, depression, and coping strategies [3,4].Thus, the quantification of the HRQoL, the disability grade, and the coping strategies is essential in treating patients after a stroke.

In recent decades, the contributions of health psychology in the field of cardiovascular diseases have gained special importance; these contributions take into account the adaptation of patients to their disease process and neurorehabilitation treatment, the adaptation to new derived lifestyles, and especially the attention to psychological needs generated by the disease status [5]. In this regard, the continuous study of psychological variables related to a patient’s health status will offer the possibility of carrying out specific interventions at a preventive level, as well as developing protective factors against diseases or strengthening healthy lifestyles [6,7].

Coping has been one of the major focuses of attention in the field of health psychology, based on the theoretical framework of Lazarus and Folkman (1984) [8], two of the main theorists of the coping study who defined it as “the constantly changing cognitive and behavioural efforts to manage the specific external or internal demands that are appraised as taxing or exceeding the resources of the person” [8]. Coping strategies, which are associated with a better HRQoL [9], have two major functions: dealing with the problem that is causing the distress (problem-focused coping) and regulating emotion (emotion-focused coping). After a stroke, patients use insufficient active problem-focused coping strategies [10].

Coping skills may be considered the key psychological resources necessary to rebuild the lives of patients disrupted by the residual deficits caused by a stroke. The possibility of adapting coping strategies that patients can use after a stroke could facilitate the design of better and more effective intervention strategies for these patients. However, to our knowledge and despite their significance, no studies have been published that explore the relationship between coping strategies in patients after an acute ischemic stroke treated with a thrombectomy. Thus, this research explores the possible relationship between coping styles, understood as the set of efforts that people make to face the problems and difficulties encountered in the environment and the subsequent stress that these situations generate, and the situation of disability that remains in patients who have suffered an acute ischemic stroke and received the most appropriate clinical treatment.

The main aim of this research was to study coping strategies, quality of life, and neurological and functional outcomes in a sample of patients at 3 months and 1 year after an ischemic stroke, who had been previously randomized and included in the Endovascular Revascularization with Solitaire Device versus Best Medical Therapy in Anterior Circulation Stroke within 8 Hours (REVASCAT) clinical trial [11] and classified in terms of the treatment received in the acute phase: either endovascular therapy (EVT, thrombectomy alone or with intravenous alteplase) or standard best medical treatment (BMT, intravenous alteplase or Stroke Unit admission).

## 2. Materials and Methods

### 2.1. Design

This was a multicenter, prospective, longitudinal, and comparative study between two groups: patients recovering from an anterior circulation proximal arterial occlusion stroke (1) treated with a Solitaire thrombectomy (EVT) or (2) treated with the standard best medical treatment (BMT) alone.

### 2.2. Participants

Patients were recruited as part of the REVASCAT clinical trial, the details of which were previously reported [11,12]. Briefly, in the REVASCAT, patients fulfilling the inclusion criteria were randomized into either of the treatment groups. This study was a sub-study of participants in the REVASCAT clinical trial. After consenting for our sub-study, 82 participants were enrolled from March 2013 to March 2015, with a follow-up until December 2016.

### 2.3. Measures and Instruments

Several sociodemographic variables were collected to study the sample characteristics. These variables included age, sex, medical history, and toxic habits (alcohol and smoking).

Brief Coping Questionnaire (Brief-COPE): The Spanish version of the Brief COPE (COPE-28) [13,14] was used to assess coping strategies at 3 months and 1 year after randomization by a researcher masked to the treatment allocation. COPE-28 is a scale that has 28 items to evaluate 14 dimensions through several questions that may be answered from “0 (I did not do this at all) to 3 (I did this a lot),” including intermediate scores. The higher the score obtained in each dimension, the greater the coping. The studied dimensions were active coping, planning, positive reframing, acceptance, humor, religion, emotional support, instrumental support, self-distraction, denial, venting, use of substances (alcohol or drugs), behavioral disengagement, and self-blame. In terms of psychometric characteristics, the alpha coefficients for this study were calculated at 3 months (α = 0.75) and 1 year (α = 0.68).

National Institute of Health Stroke Scale (NIHSS): The Spanish adapted version of the National Institute of Health Stroke Scale (NIHSS) was applied for the quantification of the neurological involvement. The NIHSS consists of 11 items, each of which provides information about neurological impairment. The score ranges on a scale between 0 and 42, 0 being evidence of normal function and 42 indicating a major neurological involvement (α =0.90) [15].

Barthel Index (BI): The Barthel Index (BI) has 10 questions about activities of daily life to assess the functional independence of stroke patients [16]. These questions are related to feeding, bathing, grooming, dressing, bowels, bladder, toilet use, transfers, mobility, and stairs, amounting to a total of 100 points. The higher the score obtained, the higher the level of functional independence (α = 0.90−0.92) [17].

Modified Rankin Scale (mRS): The modified Rankin Scale (mRS) [18,19] was used in this study to quantify patients’ functional ability after a stroke. The scale goes from 0 (asymptomatic after a stroke) to 6 (death). An mRS score between 0 and 2 indicates independence in the functional capacity after a stroke (k_w_ = 0.78−0.93) [20].

Stroke Impact Scale-16 (SIS-16): The abridged and translated version of the Stroke Impact Scale-16 (SIS-16) [21] was applied to assess the impact of impairment on patients after a stroke. This scale comprises 16 items, in which a higher score means that the patient has a better status and a lower stroke impact. An SIS-16 score between 40 and 80 is associated with less impairment and stroke impact (α = 0.83−0.90) [22].

EuroQoL (EQ-5D): The EQ-5D was designed by an interdisciplinary group of researchers from several European countries [23] and adapted to the Spanish language [24]. This questionnaire has two parts: the first one is a description of the generic health status with five dimensions (mobility, personal care, daily activities, pain/discomfort, and anxiety/depression), and the second one is the visual analog scale (EQ-VAS), where the patient quantifies their health status in a range between 0 and 100. In terms of psychometric characteristics, the alpha coefficient for this study was calculated at 3 months (α = 0.891) and 1 year (α = 0.838).

### 2.4. Ethics Statement

This study was approved by the Ethics Committee and written informed consent was obtained from all patients participating in the study. The ID protocol of the study was ICTO/2014. The study has no conflict of interest with the stent retriever commercial product (Covidien).

### 2.5. Data Analysis

Descriptive statistics were used to summarize patients’ characteristics and study different types of variables. To test the differences between groups, Student’s *t*-test and the Mann–Whitney U test were used for quantitative variables, depending on the normality of the data. A chi-squared test was used for qualitative variables. Changes in the COPE-28, mRS, and NIHSS scales at 3 months and 1 year after randomization were assessed through the Wilcoxon sign ranks test. In addition, multiple linear regression models were carried out to explain the HRQoL at 3 months and 1 year after a stroke. Statistical significance was established at *p*-value<0.05 with 95% confidence intervals (CIs).

## 3. Results

The sociodemographic and clinical-pathological characteristics of patients are presented in Table 1.The age and sex of participants were similar in both the EVT and BMT groups, and there were no significant differences between groups in all the variables except for previous strokes.

We assessed the stroke patients’ coping strategies with the COPE-28 scale after randomization and we found that active coping at 3 months and acceptance and use of emotional support at 3 months and 1 year to be the dimensions with higher scores (Table 2). Notwithstanding, the BMT group had the most average scores higher than those in the EVT group at 1 year after randomization, with the exception of the religion and humor dimensions. Importantly, the only significant differences in the scores between the patients’ groups were those recorded in active coping and acceptance (*p* = 0.03 and *p* = 0.034, respectively; Table 2).

All the scales evaluated in this study describe neurological deficits and functional capacity before and after a stroke. Although the NIHSS score at baseline was not statistically significant between groups, the score was significantly different at 24 h after randomization, showing greater neurological involvement in the EVT group (Table 3). All the participants in this study were autonomous in their activities of daily life before their stroke (BI =100) and presented favorable functional capacities, as shown by the mRS test (0–2). Although the functional status at 3 months and 1 year was greater in the EVT group, as shown by higher values in the BI, mRS, and SIS-16 scales, the difference between groups was not statistically significant (Table 3).

We performed the categorization of the NIHSS, mRS, BI, and SIS-16 scales according to the score intervals considered to indicate favorable functional and neurological statuses in order to assess which of the treated groups had a more favorable functional and neurological performance. Although the percentages of favorable evolution in all the tests were higher in the EVT group at 3 months and 1 year after randomization, only the mRS at 3 months proved to be statistically significant (Table 4).

Furthermore, the HRQoL was quantified using theEQ-5D scale at 3 months and 1 year after a stroke (Table 5). Importantly, the dimension related to pain/discomfort was the only one to show significant differences between the two groups at 3 months. Indeed, the EVT group had a higher percentage of people without pain or discomfort. In addition, the median total score obtained by the EQ-VAS was higher in this group than in the BMT group (60 vs. 50), with a clear trend toward significance (*p* = 0.053).

Finally, the results of a multivariate analysis at 3 months after a stroke showed that the variables associated with HRQoL (EQ-VAS) were the EVT (*p* = 0.038), functional status evaluated using the mRS scale (*p* < 0.001), and the pain/discomfort dimension in the EQ-5D scale (*p* < 0.001) (Table 6).

At 1 year after a stroke, the variables associated with HRQoL (EQ-VAS) were functional status assessed using the mRS scale (*p* < 0.001) and the pain dimension in the EQ-5D scale (*p*<0.001) (Table 7). In both analyses, the score on the mRS scale and pain/discomfort dimension was inversely related to the HRQoL (β = −0.739 and β = −0.330 at 3 months and β = −0.595 and β = −0.441 at 1 year). The multiple linear regression models accounted for 56% of the HRQoL variability at 3 months and 53% at 1 year, respectively (Table 6 and Table 7).

## 4. Discussion

From the evidence of the EVT efficacy, the therapeutic approach for patients with ischemic stroke has changed drastically [25]. Most previous research on the treatment of patients with ischemic stroke has focused on the improvement of patients’ neurological and functional aspects, whereas there is little research approaching the psychological sphere that studies coping strategies [26,27]. Nevertheless, the aforementioned studies do not present an accurately selected sample (as there are no selection criteria), and they are cross-sectional studies without self-reported questionnaires and with short follow-ups (<6 months) [27].

This is the first study to investigate the coping strategies of patients in the acute phase of stroke recovery based on the received treatment (EVT or BMT) in a randomized sample. Our results show that patients with ischemic stroke that were treated with either the EVT or BMT in the acute phase presented similar coping strategies at 3 months after a stroke. Nevertheless, 1 year after a stroke, patients treated with the BMT showed significantly higher scores in active coping and acceptance dimensions, which belong to problem-focused coping strategies. This suggests that patients recovering from an acute stroke after being treated with the BMT have more positive attitudes toward the condition, and therefore, take actions such as acceptance of the illness and finding solutions to improve their status [10,28].

Our results are consistent with previous research in which problem-focused coping strategies were reported more often than emotion-focused coping strategies in stroke patients [29]. It has also been observed that problem-focused, active coping strategies improve psychological outcomes in people with chronic illnesses, noting that cultivating a tendency to relate to oneself with kindness, compassion, and acceptance (self-compassion) is related to active coping strategies and can be valuable in reducing stress for those facing a chronic situation [30].

Furthermore, emotion-focused coping strategies have previously been associated with a worse stroke recovery [31,32]. Feelings of guilt, anger, and sadness are negative emotions that can arise after suffering an unexpected or uncontrollable and functionally limiting event, such as a stroke. Avoiding facing up to the illness is associated with poor results; it generates helplessness and therefore less acceptance of it. All of this can contribute to recurrent additional stress that prevents the development of healthy behaviors [29,30,32].

With regard to patients’ quality of life, the EVT group presented a higher number of patients with no pain/discomfort than the BMT group, and in turn, obtained higher scores in the analogical part of the EQ-5D scale, especially in the short term [33]. Indeed, this is in line with previous studies that indicated that patients with a chronic illness that have few but self-perceived effective coping strategies may have a more positive effect on the HRQoL than patients using other types of strategies targeting each individual’s personal resources, such as a lower sense of coherence [34]. A sense of coherence implies that the patient understands the magnitude of their illness, the degree of motivation to cope with it, and their ability to manage what this involves, which could influence coping strategies, and as a result, the impact on their quality of life [34].

Thus, encouraging patients to adopt coping strategies is a complex process because it involves the patient adopting self-management behaviors, exercising self-control of their processes and results, and actively participating in their health education, although there is limited evidence of the effect of psychosocial interventions in stroke patients that can improve the results in terms of their quality of life [35,36,37]. Therefore, effective coping should be based on using a set of coping strategies that enable stress management in the context of the illness and switching to new adaptive strategies as the situation evolves [30].

Our findings suggest that these factors should be taken into account to promote better interventions with regard to the secondary prevention for these patients and improve their HRQoL, leaving an open door for future investigations. While the technology associated with interventional strategies is improving and technical outcomes are also improving, longer-term outcomes may not be [36,37].

Although the EVT patients’ group was associated with a better HRQoL and presented a more favorable neurological and functional status compared to the BMT group, paradoxically, they did not show better coping strategies in the short term (3 months), nor in the long term (1 year) after a stroke. This could be due to the better functional and neurological outcome of the EVT patients, who showed less clinical involvement, and therefore, less perception and awareness of the illness, entailing less coping and acceptance by patients. Although this finding is revealing, it is currently difficult to discuss since there are no studies that incorporate the coping process into the assessment of the patient’s neurological and physical status, or that allow for a predictive relationship to be made between these variables.

Finally, we found the EVT to be positively associated with the HRQoL; meanwhile, pain/discomfort and functional outcomes were inversely associated at 3 months, but not at one year after having the stroke in our patient cohort [35]. This may be explained by the fact that patients undergoing the EVT reported better short- and long-term quality of life scores, and in turn, better neurological and functional statuses. A study has been published recently that reinforces how the EVT in people who have suffered a stroke has a favorable impact on the patients’ quality of life. However, coping and other variables related to their adaptation to a diagnosis of stroke have not been studied [38]. In line with this, we found similar results regarding neurological outcomes and functional capacities to those obtained in the whole population studied in the REVASCAT [39,40,41], indicating that the variables analyzed in this study can be generalized to the totality of people with acute ischemic stroke currently treated with the EVT.

### Limitations of the Study and Future Research

Although the results obtained from our study can be extrapolated to the rest of the population meeting the same criteria, we found that the neurological status led to a loss of follow-up. Hence, there was a reduction in the number of patients answering the COPE scale at 3 months and 1 year, and the other scales at 1 year. Another limitation was that no specific variables or constructs related to positive and adaptive coping strategies (such as personal abilities) and attitudes (such as resilience and self-compassion) or mediating variables (such as dispositional optimism associated with stress reduction) were studied. Therefore, future research on how people who have suffered a stroke, in addition to containing the variables studied, should include other study factors, which would allow for a greater understanding of the process and follow-up, including examining the care received and the environment of those affected.

## Figures and Tables

**Table 1:** Sociodemographic and clinical-pathological characteristics of the patients.

Sociodemografic and Clinical-Pathological Characteristics	Total (*n* = 82)	EVT (*n* = 42)	BMT (*n* = 40)	*p*-Value
Age (years)	67.60 (10.40)	66.76 (10.15)	68.47 (10.72)	0.406
Sex (male)	43 (52.4)	23 (54.8)	20 (50)	0.204
Medical history				
Psychiatric history	11 (13.4)	8 (19)	3 (7.5)	0.128
Hypertension	55 (67.1)	27 (64.3)	28 (70)	0.582
Diabetes mellitus	22 (26.8)	12 (28.6)	10 (25)	0.715
Dyslipidaemia	52 (63.4)	26 (61.9)	26 (65)	0.775
Ischemic heart disease	42 (51.2)	20 (47.6)	22 (55)	0.504
Cancer	11 (13.4)	5 (11.9)	6 (15)	0.681
Previous stroke	7 (8.5)	1 (2.4)	6 (15)	0.041
Previous TIA	3 (3.7)	1 (2.4)	2 (5)	0.337
Toxic habits				
Alcohol				0.786
Never	65 (79.3)	33 (78.6)	32 (80)	
Moderate	13 (15.9)	7 (16.7)	6 (15)	
Severe	3 (3.7)	1 (2.4)	2 (5)	
Former	1 (1.2)	1 (2.4)	0	
Smoking				0.661
Never	53 (64.6)	26 (61.9)	27 (67.5)	
Moderate	11 (13.4)	6 (14.3)	5 (12.5)	
Excessive	5 (6.1)	3 (7.1)	2 (5)	
Former	13 (15.9)	7 (16.7)	6 (15)	

Values in brackets for age are standard deviations and are percentages for all the other values of the sample. BMT: Best Medical Treatment, EVT: Endovascular Treatment.

**Table 2:** Brief Coping Questionnaire (COPE-28) Scale at 3 months and 1 year after randomization, depending on the group treatment.

COPE-28 Items	Total (*n* = 50)	COPE-28 (3 Months)	*p*-Value	COPE-28 (1 Year)	*p*-Value
Active coping	EVT: 23	3.92 [1.99]	0.267	3.63 [2.10]	0.030
Items 2 and 10	BMT: 27	4.52 [1.83]		4.74 [1.43]	
Planning	EVT: 23	2.96 [1.88]	0.465	2.79 [2.10]	0.637
Items 6 and 26	BMT: 27	3.37 [2.10]		3.07 [2.13]	
Use of emotional support	EVT: 23	3.92 [2.30]	0.753	4.25 [2.07]	0.597
Items 9 and 17	BMT: 27	4.11 [2.08]		4.56 [2.03]	
Social support	EVT: 23	3.04 [1.76]	0.195	3.21 [1.64]	0.458
Items 1 and 28	BMT: 27	3.67 [1.64]		3.59 [1.99]	
Religion	EVT: 23	1.79 [2.32]	0.574	2.50 [2.54]	0.403
Items 16 and 20	BMT: 27	1.44 [2.06]		1.93 [2.32]	
Positive reframing	EVT: 23	2.54 [2.18]	0.886	2.83 [1.99]	0.164
Items 14 and 18	BMT: 27	2.63 [2.15]		3.63 (2.02)	
Acceptance	EVT: 23	4.21 [1.79]	0.795	4.25 [1.65]	0.034
Items 3 and 21	BMT: 27	4.33 [1.62]		5.00 [1.39]	
Denial	EVT: 23	1.17 [1.63]	0.441	0.92 [1.25]	0.339
Items 5 and 13	BMT: 27	1.56 [1.91]		1.33 [1.75]	
Humor	EVT: 23	1.33 [1.93]	0.944	1.29 [1.85]	0.830
Items 7 and 19	BMT: 27	1.37 [1.82]		1.19 [1.67]	
Self-distraction	EVT: 23	3.00 [2.04]	0.499	2.75 [1.87]	0.299
Items 4 and 22	BMT: 27	2.59 [2.21]		3.33 [2.08]	
Self-blame	EVT: 23	1.29 [1.57]	0.945	1.33 [1.50]	0.548
Items 8 and 27	BMT: 27	1.26 [1.77]		1.63 [1.95]	
Behavioral disengagement	EVT: 23	1.33 [1.69]	0.728	0.71 [1.08]	0.463
Items 11 and 25	BMT: 27	1.19 [1.33]		0.96 [1.34]	
Venting	EVT: 23	1.25 [1.26]	0.496	1.54 [1.50]	0.906
Items 12 and 23	BMT: 27	1.52 [1.50]		1.59 [1.55]	
Substance abuse	EVT: 23	0.21 [0.66]	0.202	0.13 [0.61]	0.697
Items 15 and 24	BMT: 27	0.04 [0.19]		0.19 [0.48]	

Variables are represented as median [IQR].

**Table 3:** Neurological and functional outcome of patients: post-treatment, at 3 months, and 1 year after a stroke.

Neurological and Functional Scales	Total (*n* = 82)	EVT (3 Months; *n* = 42) (1 Year; *n* = 35)	BMT (3 Months; *n* = 35) (1 Year; *n* = 31)	*p*-Value
Baseline NIHSS	16 [13−19]	15 [11−19]	17 [14−19]	0.225
NIHSS 24 h	10 [4−15]	6 [3−13]	11 [7−17]	0.013
NIHSS 3 months	7 [1−11]	2 [1−12]	5 [3−11]	0.071
NIHSS 1 year	5 [1−7]	4 [0−8]	6 [1−7]	0.484
Previous BI	100	100	100	1.000
BI 3 months	80 [10−98]	80 [13−100]	70 [10−95]	0.184
BI 1 year	76 [60−100]	78 [65−100]	74 [60−95]	0.336
Previous mRS	0 [0−0]	0 [0−0]	0 [0−1]	0.355
mRS 3 months	3 [2−5]	2 [2−5]	3 [2−5]	0.178
mRS 1 year	3 [2−3]	2 [2−3]	3 [2−3]	0.536
SIS-16 3 months	53 [21−73]	59 [22−75]	48 [16−70]	0.269
SIS-16 1 year	57 [46−76]	60 [53−78]	54 [37−71]	0.178

Variables are represented with median [IQR]. BI: Barthel Index, mRS: Modified Rankin Scale, NIHSS: National Institute of Health Stroke Scale, SIS-16: Stroke Impact Scale-16.

**Table 4:** The number of patients included in the favorable functional and neurological category of the evaluated scales at 3 months and 1 year after a stroke.

Neurological and Functional Scales	3 Months			1 Year		
	EVT (*n* = 42)	BMT (*n* = 40)	*p*-Value	EVT (*n* = 35)	BMT (*n* = 31)	*p*-Value
NIHSS [0–2]	22 (81.5)	8 (61.5)	0.314	17 (60.7)	14 (53.8)	0.876
mRS [0–2]	21 (50)	11 (27.5)	0.029	20 (47.6)	11 (27.5)	0.119
BI [95–100]	16 (43.6)	12 (35.3)	0.881	17 (65.4)	13 (52)	0.332
SIS-16 [40–80]	27 (65.9)	24 (60)	0.585	28 (80)	23 (74.2)	0.574

Variables are represented with number of cases (percentage).

**Table 5:** The health-related quality of life (HRQoL) perceived by participants at 3 months and 1 year after a stroke.

HRQoL	EVT 3 Months (*n* = 40)	BMT 3 Months (*n* = 36)	*p*-Value	EVT 1 Year (*n* = 35)	BMT 1 Year (*n* = 31)	*p*-Value
Mobility			0.204			0.635
I have no trouble walking	21 (52.5)	12 (33.3)		18 (51.4)	13 (41.9)	
I have some trouble walking	11 (27.5)	16 (44.4)		12 (34.3)	14 (45.2)	
I have to stay in bed	8 (20)	8 (22.2)		5 (14.3)	4 (12.9)	
Personal care			0.129			0.514
I have no problems with personal care	21 (52.5)	12 (33.3)		20 (57.1)	14 (45.2)	
I have some difficulty in washing or dressing myself	7 (17.5)	13 (36.1)		7 (20)	10 (32.3)	
I am unable to wash or dress myself	12 (30)	11 (30.6)		8 (22.9)	7 (22.6)	
Daily activities			0.645			0.792
I have no problems	12 (30)	8 (22.2)		10 (28.6)	8 (25.8)	
I have some problems	15 (37.5)	13 (36.1)		13 (37.1)	13 (41.9)	
I am unable to do daily activities	13 (32.5)	15 (41.7)		12 (34.3)	10 (32.3)	
Pain/discomfort			0.012			0.426
I have no pain or discomfort	22 (55)	10 (27.8)		17 (48.6)	11 (35.5)	
I have moderate pain or discomfort	11 (27.5)	22 (61.1)		14 (40)	13 (41.9)	
I have a lot of pain or discomfort	7 (17.5)	4 (11.1)		4 (11.4)	7 (22.6)	
Anxiety/depression			0.838			0.797
I am not anxious or depressed	13 (32.5)	10 (27.8)		12 (34.3)	12 (38.7)	
I am moderately anxious or depressed	20 (50)	18 (50)		18 (51.4)	15 (48.4)	
I am very anxious or depressed	7 (17.5)	8 (22.2)		5 (14.3)	4 (12.9)	
EQ-5D index	0.760 (0.177)	0.692 (0.150)	0.078	0.763 (0.160)	0.731 (0.167)	0.440
EQ-VAS (0–100)	60 [40–75]	50 [21–70]	0.053	58 [50–75]	56 [40–80]	0.707

The EuroQoL visual analog scale (EQ-VAS) is represented as median [IQR], while the rest of the variables are shown with the number of cases (percentage).

**Table 6:** Variables associated with the HRQoL (EQ-VAS) at 3 months after a stroke.

Variable	B	Standard Error	β	*p*-Value	(CI 95%)	
					Lower Limit	Upper Limit
Constant	1.013	0.065		0.000	0.883	1.143
Age	0.001	0.001	0.049	0.400	−0.001	0.003
Sex	−0.015	0.018	0.046	0.405	−0.052	0.021
Treatment (EVT)	0.039	0.019	0.118	0.038	0.002	0.077
NIHSS	0.003	0.002	0.129	0.110	−0.001	0.007
mRS	−0.078	0.009	−0.739	0.000	−0.096	−0.061
Pain/discomfort dimension (EQ-5D)	−0.078	0.016	−0.330	0.000	−0.111	−0.046

Adjusted R^2^ = 0.563.

**Table 7:** Variables associated with the HRQoL (EQ-VAS) at 1 year after a stroke.

Variable	B	Standard Error	β	*p*-Value	(CI 95%)	
					Lower Limit	Upper Limit
Constant	1.063	0.066		0.000	0.930	1.195
Age	0.001	0.001	0.059	0.319	−0.001	0.003
Sex	−0.016	0.019	−0.050	0.393	−0.054	0.021
Treatment (EVT)	−0.131	3.566	−0.003	0.971	−7.266	7.004
NIHSS	−0.001	0.003	−0.023	0.805	−0.006	0.004
mRS	−0.070	0.011	−0.595	0.000	−0.092	−0.048
Pain/discomfort dimension (EQ-5D)	−0.099	0.014	−0.441	0.000	−0.127	−0.070

Adjusted R^2^ = 0.536.
